# Attitudes and beliefs of healthcare workers towards obese persons in occupied Palestinian territories: a cross-sectional study

**DOI:** 10.1186/s12889-026-26224-8

**Published:** 2026-01-27

**Authors:** Sana Al-Aqqad, Nihal Natour, Amera Shaheen, Saja Elewi, Eman Shawahna, Sajeda Hamadi, Wafaa Najjar, Siwar Hajj, Hoor Abatli

**Affiliations:** 1 Division of Nursing and Midwifery- Ibn Sina College for Health Professions, Nablus University for Vocational & Technical Education (NUVTE), P.O. Box 110, Nablus, West Bank Palestine; 2 Department of Public Health, School of Medicine, An-Najah National University, Nablus, Palestine; 3 Faculty of Medicine and Health Sciences, An-Najah National University, Nablus, Palestine; 4 Clinical Research in Health Science- Faculty of Graduate Studies, An-Najah National University, Nablus, Palestine

**Keywords:** Obesity, Healthcare workers, Stigma, Weight, Attitudes, Beliefs

## Abstract

**Background:**

Obesity is a growing public health concern in Palestine and worldwide, associated with significant health risks and costs. Obesity stigma includes negative attitudes, stereotypes, and discrimination toward individuals based on their weight and is common in healthcare settings. This study aims to assess healthcare workers’ attitudes and beliefs toward patients with obesity in the occupied Palestinian territories.

**Methods:**

This cross-sectional study was conducted among healthcare workers in hospitals and outpatient facilities in the occupied Palestinian territories. Attitudes toward obesity were assessed using the Attitudes Toward Obese Persons (ATOP-20) scale, while beliefs about obesity were measured using the Beliefs About Obese Persons (BAOP-8) scale.

**Results:**

A total of 103 healthcare workers participated in the study (mean age: 32.1 ± 12.0 years; 60% female). Overall attitudes toward obesity were predominantly negative across professional roles. More than half of the participants attributed obesity to biological factors, lack of love, overeating, or food addiction. Female healthcare workers (-3.4 (-5.1, -1.7), *p* = 0.033) and obese participants (0.47 (0.31,0.63), *p* = 0.006) and the ones with BSc education (5.7 (3.1, 8.3), *p* = 0.029) demonstrated significantly more negative beliefs and less positive attitudes toward obesity (*p* < 0.05). In contrast, years of professional experience, department, and number of patients treated were not significantly associated with attitudes.

**Conclusion:**

The study suggests healthcare workers may hold unconscious weight biases, especially females with less education, due to misconceptions about obesity. This underscores the need for education and awareness programs to reduce bias, promote understanding, and support compassionate, patient-centered care.

## Background

Obesity is a complex health condition caused by excess body fat that harms both physical [1, 2] and mental health [3]. It also results in high healthcare costs for countries, regardless of income level [4]. It is a global health problem, including in developed and developing countries, with particularly higher rates in richer countries [5]. Obesity has recently become a problem in poorer countries, including Palestine [6]. A recent survey in Palestine showed that in the Gaza Strip, 23.6% of people are overweight and 19.5% are obese, while in the West Bank, 26.1% are overweight or obese [7]. People with obesity face greater risks of health problems like diabetes, high blood pressure, high cholesterol, heart disease, lung issues, and other related illnesses [8, 9]. The economic burden is also significant, with some rich countries like the US spending over 200 billion dollars annually just on medical care for obese adults [10].

Obesity stigma refers to negative attitudes toward individuals based on their weight [11]. It can manifest as negative feelings, stereotypes, prejudice, and unfair treatment [2]. Common stereotypes portray overweight or obese people as lazy, greedy, lacking willpower and discipline, and suggest they are unable to improve their health or unwilling to follow medical advice. Many perceive individuals with obesity as personally responsible for their extra weight. This stigma can lead to overt discrimination and biased behavior. Obesity stigma can be explicit, involving consciously held negative beliefs that individuals can report on surveys, or implicit, involving unconscious negative attitudes that occur automatically and outside awareness. Implicit bias can be measured using tools such as implicit association tests. Exposure to stigma may also lead individuals to internalize these negative beliefs, blaming themselves, adopting harmful stereotypes, and experiencing shame, which can negatively affect self-perception and increase acceptance of unfair treatment [12].

There was an increase in weight discrimination from 7% in 1995–1996 to 12% in 2004–2006, according to data from the USA [13]. Its prevalence now matches that of discrimination based on race or age, yet it does not have the same social and legal protections [14]. Tackling weight bias is essential for creating fair protections and fostering a society that respects the rights and dignity of all individuals, regardless of body size [15].

Weight stigma often stems from cultural beliefs that people with obesity tend to overeat, binge eat, and lack the motivation to change their habits. These views see obesity as a personal fault, ignoring the complex health factors involved [16]. Social media has made these perceptions worse; many influencers promote ideal images of a perfect body and lifestyle. This often reinforces negative stereotypes about obesity and sharpens public bias [17].

Obesity is common worldwide, yet many people with obesity face discrimination in places like schools, workplaces, and healthcare settings. Probably the main causes of discrimination are that healthcare workers have internalized societal negative attitudes towards obese people. It includes discriminating against people with a body mass index (BMI) over 35 at work [18], negative treatment from healthcare workers [19], including individuals reported receiving insensitive or judgmental comments from healthcare staff [20]. Studies show that primary care doctors tend to spend less time with obese patients, often because they see these patients as noncompliant [21]. Healthcare providers’ negative attitudes can stop them from giving proper support and caring treatment. This bias worsens the health care these patients receive, making it harder for them to seek help [22]. As a result, many avoid regular checkups and screenings, which can lead to worse health outcomes and less use of important health services [23].

The common view is that labeling someone as obese will encourage them to lose weight [24]. However, studies show that weight stigma causes serious and long-lasting harm to both physical and mental health [15]. People with obesity often face this stigma, which damages their confidence in their ability to manage their weight [2]. Paradoxically, studies show that healthcare providers, including doctors and nurses, can hold both conscious and unconscious biases against people with obesity. This research aims to evaluate healthcare workers’ attitudes and beliefs toward obesity in hospitals and outpatient facilities in the occupied Palestinian territories, and to understand which factors contribute to negative attitudes.

## Methods

### Study design and setting

A cross-sectional analytical study was conducted among healthcare workers in hospitals and outpatient settings in the occupied Palestinian territories. The study assessed attitudes toward people with obesity and beliefs about obesity, explored variations by socio-demographic and professional characteristics, and identified independent predictors of attitudes. Reporting followed the STROBE guidelines for cross-sectional studies [25].

### Target population and eligibility

The study targeted licensed healthcare workers providing direct patient care, including consultants, specialists, residents, interns, nurses, and allied health professionals. Eligible participants were aged 18 years or older, actively employed in a healthcare setting, and had provided informed consent. Exclusion criteria included students not providing direct care, those who declined participation, and individuals whose questionnaires contained more than 20% missing responses on any primary scale.

### Sampling strategy and sample size

Health workers in Palestine are around 37,000, according to RAOSOFT website. Sample calculation based on a margin of error of 5% and confidence level of 95%, the total number needed is 381; however, due to the war in Gaza and limited funding for this work, we used a convenience sample of 103. This involved recruiting health care workers from many institutions and private clinics in Palestine based on university alumni social media platforms. The justification for the chosen sample size is rooted in the exploratory nature of the study, which aimed to investigate attitudes and beliefs among specific subgroups of healthcare professionals, including pharmacists, nutritionists, and interns. This sample size is deemed adequate for identifying significant trends and establishing a baseline for future research. Furthermore, the multiple regression model, which included five primary predictors, aligns with the general guideline of having at least 10–20 observations per predictor. This ensures the stability of the model and the reliability of the significant predictors identified, such as Body Mass Index (BMI), gender, and education.

### Missing data handling

The study on healthcare workers, with a sample size ranging from 101 to 103 participants, demonstrated a high level of data completeness, we ensured that only participants with full responses for the relevant variables were included in the primary analyses, such as the multiple regression model and group comparisons. The individual item response rates for the ATOP 20 and BAOP 8 scales indicated minimal missingness.

### Variables

Healthcare workers’ attitudes toward obesity were evaluated using the Attitudes Toward Obese Persons (ATOP-20) scale (higher scores mean more positive attitudes). The ATOP-20 scale is a 20-item, 6-point Likert (strongly disagree, strongly agree), (1 strongly disagree to 6 strongly agree). Negatively worded items were reverse-coded, so higher totals indicate more positive attitudes (range 20–120) [26]. Beliefs about obesity were assessed among healthcare workers using the Beliefs About Obese Persons (BAOP-8) scale (where higher scores mean more negative beliefs or that obesity is controllable). The BAOP-8scale is an 8-item, 6-point Likert scale assessing causal beliefs (1 strongly disagree to 6 strongly agree). A scale 1-less than 1.67 was considered low, 1.67- less than 2.34 was considered medium, and 2.34 or higher was considered high [27]. Higher scores represented more negative beliefs. Items were coded so higher totals reflect more negative beliefs/greater attribution to controllable causes (range 8–48); Explanatory variables were collected through the questionnaire, including demographics (age, gender), BMI (kg/m²) calculated from weight/height, education, role, department, years of experience, and the number of typical patients/days.

### Translation, customization, and pilot testing

The Arabic versions of the ATOP and BAOP scales underwent recommended cross-cultural adaptation, including expert panel review to ensure semantic, idiomatic, experiential, and conceptual equivalence. A pilot test was conducted with approximately 20 healthcare workers, followed by consensus-based reconciliation. Minor wording adjustments were made without altering the meaning of the constructs [28].

### Data collection procedure

The data were collected from the healthcare workers by using an anonymous electronic questionnaire (self-administered Google Form questionnaire), which was open for responses between July 1 and July 31, 2025. The electronic format allowed participants to respond privately, thereby maintaining confidentiality and fostering a safe environment for disclosure.

An electronic informed consent form was presented at the beginning of the survey, and participants were required to indicate their consent before proceeding. We reached out to the group based on university social media platforms for previous students in health health-related field, and we also approached some healthcare facilities through volunteers who approached employees in person. The participants were sent a Google Form questionnaire using electronic messages on Facebook or the WhatsApp application. We desired to have responses from many workers in health care, including physicians, pharmacists, nurses, physiotherapists, dietitians, and even reception employees in hospitals.

### Statistical analysis

Data analysis was performed using SPSS version 21 (with a two-tailed significance level set at α = 0.05). Descriptive statistics (such as age, BMI, scale scores) were displayed and summarized with means and standard deviations (± SD), while categorical variables were presented as counts with percentages.

The Reliability and internal consistency were assessed using Cronbach’s α, conducted item–total correlations, and “α if item deleted”. Interpretation based on contemporary recommendations that values between 0.70 and 0.95 indicate acceptable consistency, depending on the research purpose [29]. Based on a pilot study performed on 20 study participants, Cronbach’s alpha for ATOP-20 was 0.8 and for BAOP-8 was 0.8, which matches other populations. Independent samples were examined using *t*-tests for binary variables (e.g., gender) and one-way ANOVA for categorical variables (education, role, department, experience, and workload).

Pearson correlation coefficients were used to assess associations among attitudes (ATOP-20 scale), beliefs (BAOP-8 scale), age, and BMI.

The primary multivariable analysis modeled ATOP-20 total scores as the dependent variable, with predictors including age, gender, BMI, education, BAOP-8 scores,. Homoscedasticity, normality were checked, and residual plots were normally distributed and adequately spread. Multicollinearity we assessed using tolerance and variance inflation factor (VIF), and was acceptable.

### Bias minimization techniques

To minimize selection bias, participants were recruited across multiple sites, departments, and work shifts to ensure diversity and representativeness of the sample. Information bias was mitigated by using validated measurement instruments, standardized administration procedures, and assurances of anonymity. In order to mitigate potential confounding, relevant covariates were predetermined and added to multivariable models. Diagnostic tests were then conducted to evaluate the adequacy of adjustment.

### Ethical considerations

The study was conducted under the principles of the Declaration of Helsinki. Ethical approval was granted by the Institutional Review Board (IRB) at An-Najah National University.

## Results

A total of 103 healthcare workers participated in the study. The mean age was 32.1 ± 12.0 years, and the mean BMI was 26.3 ± 5.7 kg/m². Most participants (70.9%) held a bachelor of science (BSc) degree, and 60% were female. Over half (60.8%) reported seeing fewer than 20 patients per day. The sample included specialists, interns, and nurses, with 31% having more than 10 years of professional experience. Table 1 summarizes the characteristics of the study population.

**Table 1:** Characteristics of the study sample

Variable	Mean± SD or n (%)
Age (years)	32.1±12.0
BMI (Kg/m2)	26.3±5.7
Gender	
Male	40 (39.6%)
Female	61 (60.3%)
Education	
College	6 (5.8%)
BSc	73 (70.9%)
Graduate	15 (14.6%)
Specialty	9 (8.7%)
Number of patients seen/day	
< 10	32 (31.4%)
10-19	30 (29.4%)
20-29	12 (11.8%)
≥ 30	28 (27.5%)
Department	
Surgery	6 (5.9%)
Internal medicine	14 (13.7%)
Outpatient settings/Clinics: (Pharmacy & nutrition departments)	82 (80.4%)
Work as	
Consultant	3 (2.9%)
Specialist	18 (17.5%)
Resident	7 (6.8%)
Intern	20 (19.4%)
Nurse	20 (19.4%)
Pharmacists & nutritionists	35 (34.0%)
Work Experience	
< 5 years	47 (45.6%)
5- Less than 10 years	19 (18.4%)
10-less than 15 years	7 (6.8%)
15-less than 20 years	12 (11.7%)
≥ 20 years	18 (17.5%)

*SD* Standard deviation, *BSc* Bachelor of Science, *MBI* Body Mass Index

Table 2 presents the responses to the attitudes toward obese people (ATOP) questionnaire. The statement “Obese people are usually sociable” received the highest mean score (4.96 ± 1.05). Participants showed high agreement with the Idea that “Severely obese people are usually untidy” (4.11 ± 1.54). The lowest scores were for “Obese workers cannot be as successful as other workers” (1.90 ± 1.27) and “Most people feel uncomfortable when they associate with obese people” (2.05 ± 1.23), suggesting these particular stigmas were less prevalent.

**Table 2:** Percentage of Response to (Attitude towards obesity) ATOP 20 Questionnaire

	Strongly disagree	Moderately disagree	Slightly disagree	Slightly agree	Moderately agree	Strongly agree	Likert scale	Level
1. Obese people are as happy as non-obese people.	25 (24.3%)	25 (24.3%)	14 (13.6%)	13 (12.6%)	21 (20.4%)	5 (4.9%)	2.95 ± 1.61	High
2. Most obese people feel that they are not as good as other people.	10 (9.7%)	14 (13.6%)	17 (16.5%)	29 (28.1%)	24 (23.3%)	9 (8.7%)	3.68 ± 1.44	High
3. Most obese people are more self-conscious than other people	22 (21.3%)	22 (21.3%)	20 (19.4%)	20 (19.4%)	16 (15.5%)	3 (2.9%)	2.95 ± 1.47	High
4. Obese workers cannot be as successful as other workers.	55 (53.3%)	25 (24.3%)	10 (9.7%)	6 (5.8%)	5 (4.9%)	2 (1.9%)	1.90 ± 1.27	Medium
5. Most non-obese people would not want to marry anyone who is obese.	10 (9.7%)	19 (18.4%)	17 (16.5%)	25 (24.3%)	26 (25.2%)	6 (5.8%)	3.54 ± 1.44	High
6. Severely obese people are usually untidy.	9 (8.7%)	11 (10.7%)	7 (6.8%)	31 (30%)	22 (21.4%)	23 (22.3%)	4.11 ± 1.54	High
7. Obese people are usually sociable	0 (0%)	5 (4.9%)	12 (11.7%)	29 (28.2%)	41 (39.8%)	16 (15.5%)	4.96 ± 1.05	High
8. Obese people are not dissatisfied with themselves	4 (4%)	18 (17.8%)	33 (32.7%)	25 (24.8%)	15 (14.9%)	6 (5.9%)	3.49 ± 1.51	High
9. Obese people are just as confident as other people.	7 (6.9%)	18 (17.6%)	21 (20.6%)	25 (24.5%)	21 (20.6%)	10 (9.8%)	3.38 ± 1.46	High
10. Most people feel uncomfortable when they associate with obese people.	50 (48.5%)	18 (17.5%)	20 (19.4%)	10 (9.7%)	5 (4.9%)	0 (0%)	2.05 ± 1.23	Medium
11. Obese people are often less aggressive than non-obese people.	14 (13.9%)	14 (13.9%)	19 (18.8%)	26 (25.7%)	19 (18.8%)	9 (8.9%)	3.49 ± 1.51	High
12. Most obese people have different personalities from non-obese people.	16 (15.7%)	13 (12.7%)	18 (17.6%)	31 (30.4%)	19 (18.6%)	5 (4.9%)	3.38 ± 1.46	High
13. Very few obese people are ashamed of their weight.	8 (7.8%)	15 (14.7%)	19 (18.6%)	29 (28.4%)	27 (26.5%)	4 (3.9%)	3.63 ± 1.33	High
14. Most obese people resent normal-weight people.	18 (17.4%)	25 (24.3%)	18 (17.5%)	28 (27.1%)	13 (12.6%)	1 (0.01%)	2.96 ± 1.35	High
15. Obese people are more emotional than non-obese people.	7 (6.9%)	9 (8.8%)	18 (17.6%)	28 (27.5%)	28 (27.5%)	12 (11.8%)	3.95 ± 1.38	High
16. Obese people should not expect to lead normal lives.	26 (25.2%)	19 (18.4%)	22 (21.4%)	25 (24.3%)	8 (7.8%)	3 (2.9%)	2.80 ± 1.41	High
17. Obese people are just as healthy as non-obese people.	44 (42.7%)	28 (27.2%)	17 (16.5%)	9 (8.7%)	3 (2.9%)	2 (1.9%)	2.08 ± 1.23	High
18. Obese people are just as attractive as non-obese people.	14 (13.6%)	16 (15.5%)	30 (29.1%)	18 (17.5%)	17 (16.5%)	8 (7.8%)	3.31 ± 1.47	High
19. Obese people tend to have family problems	19 (18.4%)	14 (13.6%)	34 (33.0%)	20 (19.4%)	14 (13.6%)	2 (1.9%)	3.01 ± 1.34	High
20. One of the worst things that could happen to a person would be for him to become obese	20 (19.6%)	10 (9.8%)	19 (18.6%)	24 (23.5%)	18 (17.6%)	11 (10.7%)	3.42 ± 1.63	High

Table 3 summarizes the main components of beliefs about obese people. The strongest beliefs were that obesity is caused by poor eating habits (4.82 ± 1.16) and overeating (4.56 ± 1.26). There was significant agreement that a lack of exercise (4.23 ± 1.33) and biological disorders (4.20 ± 1.27) are major contributors to obesity.

**Table 3:** Percentage of response to the belief of obese people towards obesity (BAOP 8)

	Strongly disagree	Moderately disagree	Slightly disagree	Slightly agree	Moderately agree	Strongly agree	Likert scale	Level
1. Obesity often occurs when eating is used as a form of compensation for a lack of love or attention.	9 (8.8%)	19 (18.6%)	12 (11.8%)	37 (36.3%)	20 (19.6%)	5 (4.9%)	3.54 ± 1.36	High
2. In many cases, obesity is the result of a biological disorder.	4 (3.9%)	8 (7.8%)	10 (9.8%)	37 (36.3%)	28 (27.5%)	15 (14.7%)	4.20 ± 1.27	High
3. Obesity is usually caused by overeating	4 (3.9%)	3 (2.9%)	11 (10.8%)	21 (20.6%)	40 (39.2%)	23 (22.5%)	4.56 ± 1.26	High
4. Most obese people cause their problem by not getting enough exercise.	5 (4.9%)	6 (5.9%)	16 (15.7%)	26 (25.5%)	32 (31.4%)	17 (16.7%)	4.23 ± 1.33	High
5-Most obese people eat more than non-obese people	7 (6.9%)	12 (11.8%)	12 (11.8%)	19 (18.6%)	31 (30.4%)	21 (20.6%)	4.16 + ± 1.53	High
6. The majority of obese people have poor eating habits that lead to their obesity	3 (2.9%)	2 (1.9%)	7 (6.8%)	17 (16.5%)	44 (42.7%)	30 (29.1%)	4.82 ± 1.16	High
7. Obesity is rarely caused by a lack of willpower	17 (16.5%)	19 (18.4%)	22 (21.3%)	16 (15.5%)	21 (20.3%)	8 (7.8%)	3.28 ± 1.57	High
8. People can be addicted to food, just as others are addicted to drugs, and these people usually become obese.	4 (3.9%)	8 (7.8%)	6 (5.8%)	14 (13.6%)	33 (32.0%)	38 (36.9%)	3.73 ± 1.42	High

Table 4 Compares ATOP and BAOP scores across study variables. Females had significantly higher negative beliefs about obese people (26.8 ± 4.0) compared to males (22.9 ± 8.8, *p* = 0.003). Education level was not significantly associated with ATOP-20 or BAOP-8 scores.

**Table 4:** Comparison of the ATOP 20 score and BAOP 8 score between study groups

Variable	ATOP 20	BAOP 8
Gender		
Male	60.9±7.9	22.9±8.8
Female	61.2±7.9	26.8±4.0
	T -0.2, p=0.84	T=-3.0, P=0.003
Education		
College	60.3±6.5	26.1±6.7
BSc	62.2±7.7	24.1±8.0
Graduate	58.3±8.0	25.8±4.4
Specialty	61.2±5.6	25.6±6.0
	F=1.6, P=0.195	F=0.16, P=0.93
Number of patients		
Less than 10	60.0±7.1	26.1±6.7
11- Less than 20	59.8±8.7	24.1±7.9
20-less than 30	62.2±11.2	25.8±4.4
30 or more	63.7±10.5	25.6±6.0
	F=1.05, p=0.37	F=0.47, P=0.70
Department		
Surgery	63.3±8.1	23.0±8.9
Internal medicine	63.5±5.5	24.5±7.6
Outpatient settings/Clinics: (Pharmacy & nutrition departments)	60.7±8.2	25.6±6.4
	F=1.5, P=0.228	F=0.56, P=0.58
Work as		
Consultant	64.3±5.0	18.3±9.2
Specialist	60.2±9.7	23.0±7.6
Resident	61.3±7.0	26.5±6.1
Intern	61.9±8.2	24.4±5.4
Nurse	59.9±8.0	27.0±6.3
Pharmacists & nutritionists	63.7±7.6	26.7±7.8
	F=0.44, P=.82	F=1.8, p=0.12
Experience		
< 5 years	61.2±8.4	25.5±6.2
5-10 years	58.4±9.2	25.8±7.6
10-15 years	61.4±2.7	22.7±7.2
15-20 years	65.5±5.1	26.9±5.9
>20 years	61.2±7.9	27.0±6.3
	F=0.61, P=0.66	24.6±7.2
		F=0.52, p=0.72

*ATOP* Attitudes Toward Obese Persons, *BAOP* Beliefs of obese people towards obesity

Table 5 The regression model (R² = 0.185) identified the factors that significantly predict attitudes toward obese persons. Higher BMI was a significant negative predictor of ATOP 20 scores (B = 0.47, *p* = 0.006), suggesting that individuals with higher BMI may have more negative attitudes toward others with obesity. Gender was a significant predictor (*p* = 0.033), with males showing a negative association compared to females. Having a BSc degree was also a significant predictor (*p* = 0.029) compared to the reference group. Age and BAOP 8 scores did not significantly predict ATOP 20 scores in this model.

**Table 5:** A multiple regression of Predictors of the ATOP 20 score

Variable	B (95% CI)	T-value	P-value
Age	0.10 (0.02, 0.18)	1.2	0.22
Gender (male vs female)	-3.4 (-5.1, -1.7)	-2.2	0.033
Education			
College	4.9 (0.7, 9.1)	1.2	0.24
BSc	5.7 (3.1, 8.3)	2.2	0.029
Graduate	-0.4 (-3.5, 2.8)	-0.1	0.91
Specialty	ref		
BMI	0.47 (0.31,0.63)	2.8	0.006
BAOP 8 score	0.18 (0.07, 0.29)	1.50	0.124

*BMI* Body Mass Index, *BAOP* Belief of obese people towards obesity (R-squared=0.185)

### Comparison of attitude towards obese people and belief towards obese people according to BMI category

**Fig. 1:**
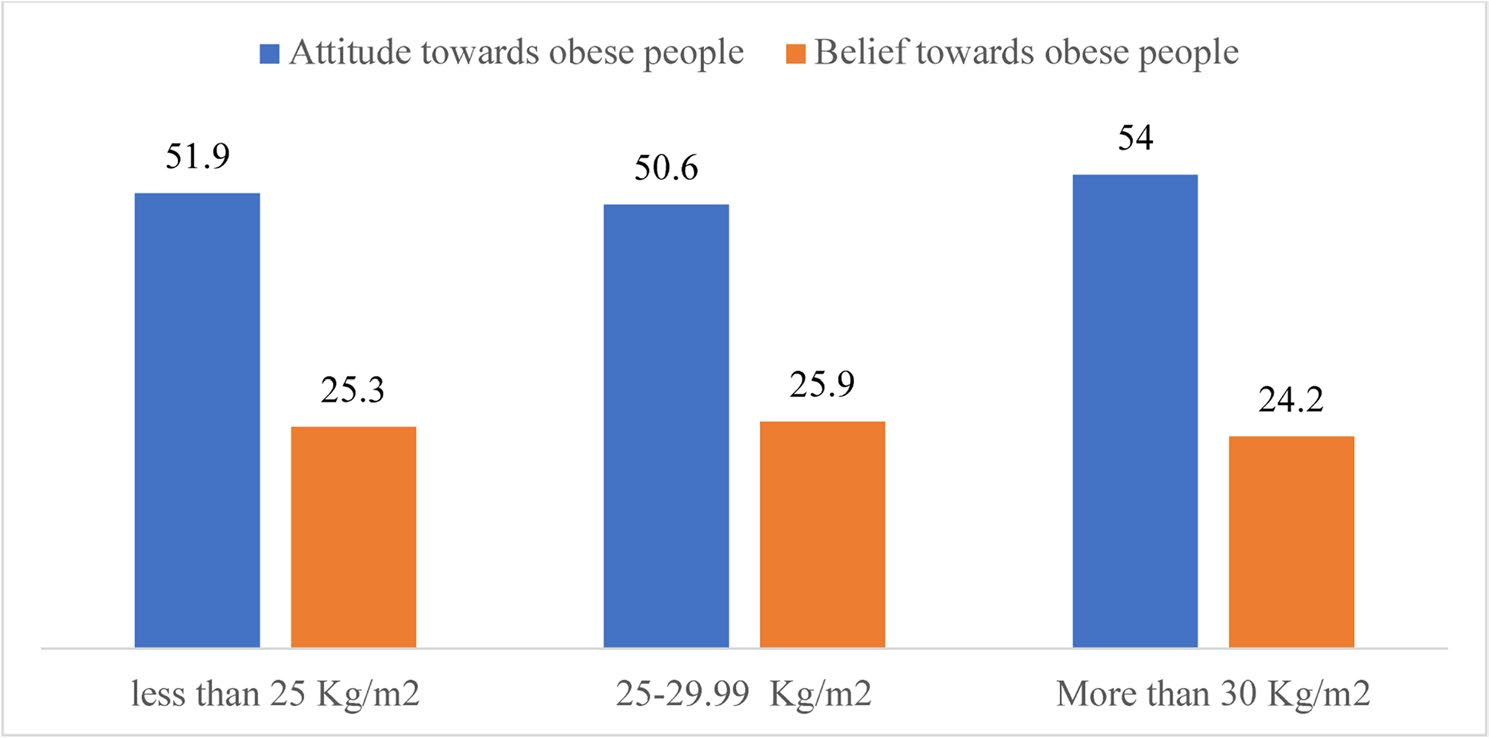
Comparison of Attitude towards obese people and belief towards obese people according to BMI category

The median scores for healthcare professionals’ attitudes toward obese people (ATOP) and beliefs about obese people (BAOP) across different BMI categories were compared in Fig. 1. Those with higher BMI were more likely to exhibit slightly more favorable attitudes and less negative beliefs regarding individuals with obesity; however, no statistically significant differences were found among the BMI groups (*p* > 0.05).

## Discussion

In this study, healthcare workers, including nurses, interns, specialists, consultants, and residents, demonstrated negative attitudes toward obesity, as measured by the ATOP-20 scale. This finding is consistent with previous research involving healthcare professionals. A literature review of nurses, despite limitations in sampling and measurement, also reported a persistent negative attitude toward obesity [30]. Similarly, a 2005 study in France among general practitioners found that approximately 30% of participants held negative views toward overweight and obese patients [31], and another study among primary care physicians reported that over 50% exhibited negative attitudes toward obesity [32]. Our results are also in line with studies assessing attitudes among health-related students. For example, a 2009 study in South Africa found physiotherapy students displayed negative attitudes toward obesity [33], and similar patterns were observed among dietetics students [34]. Medical students surveyed on this topic likewise showed a prevalence of negative attitudes [35]. Nurse students were similarly affected, as evidenced by a 2009 study reporting comparable results [36]. This consistent pattern reflects the presence of unconscious weight bias, which persists despite significant efforts to maintain neutrality and treat patients fairly [37–40]. Such bias is often rooted in cultural norms and longstanding habits, and it can influence individuals across all populations, regardless of their educational background [41].

Our study found that over half of the participants attributed obesity primarily to biological factors, lack of affection, overeating, and food addiction, as measured by the BOAP-8 scale. These results contrast with earlier research, which emphasized environmental and policy-related contributors—such as food availability, taxation, labeling, and advertising, as well as individual factors like willpower, motivation, and time constraints [42, 43]. Among healthcare professionals, previous studies have identified common barriers to obesity management, including limited consultation time, low patient motivation, and competing clinical priorities [44]. In contrast, research using alternative belief instruments has often attributed obesity to behavioral rather than physiological or psychological causes [45]. These differences likely reflect methodological variation, as different instruments constrain participants’ responses to predefined domains. Studies employing the BOAP instrument in various countries have generally reported moderate to negative beliefs regarding patient control over obesity [46–48]. This is broadly consistent with our findings. However, direct comparisons remain limited due to variations in scoring methods and analytical approaches. Overall, these inconsistencies highlight the challenges of cross-study comparisons and underscore the need for greater standardization of measurement tools and analytical frameworks when assessing healthcare professionals’ beliefs about obesity [49].

Importantly, regression analyses in this study indicated that female healthcare workers, individuals with lower educational levels (e.g., a bachelor’s degree), and obese professionals tended to hold more negative attitudes about obesity; these differences were statistically significant. Conversely, those with college or specialized training appeared to have more negative attitudes, this was statistically significant. These findings align with international evidence showing that advanced education and exposure to obesity-specific curricula can reduce prejudice [50, 51]. However, the persistence of negative stereotypes across different experience levels suggests that education alone may not be sufficient to eliminate weight stigma. Unconscious biases are likely to require targeted interventions, such as empathy training and structured anti-stigma programs, as highlighted in previous studies [52, 53].

Weight stigma in healthcare has significant consequences. Previous studies have shown that patients who perceive bias from healthcare providers are less likely to seek preventive care, more likely to delay treatment, and more likely to experience psychological distress and eating disorders [21, 54, 55]. Therefore, the findings of this study underscore the urgent need to address obesity stigma at both educational and institutional levels, not only to enhance patient-provider relationships but also to reduce health disparities and improve clinical outcomes.

### Limitations

Several limitations of this study should be noted. First, the cross-sectional design prevents any causal conclusions regarding the relationship between demographic factors and attitudes toward obesity. Second, the sample was drawn from a limited number of healthcare institutions, which restricts the generalizability of the findings to other regions or healthcare systems. Third, the use of self-administered questionnaires may have introduced social desirability bias, given the sensitivity of the topic. Future research should consider longitudinal, mixed-methods designs, employ standardized assessment tools, and include more diverse healthcare settings across the Arab world and beyond. One more point, responses to attitude and belief questionnaires could be influenced by social desirability bias. The issue of stigmatizing obese patients could be very sensitive to address among health workers, and so they may have responded in a biased way to cover some negative feelings.

### Recommendations

Based on the findings of this study, it is recommended that educational and awareness programs be implemented for healthcare workers to reduce weight bias and negative attitudes toward obesity. Such interventions could improve the quality of care and foster more supportive clinical environments for individuals affected by obesity. Additionally, further research is needed to explore healthcare professionals’ beliefs and attitudes toward obesity across the broader Arab world, as cultural and contextual factors may influence perceptions and practices. Finally, efforts should be made to develop a standardized tool for assessing beliefs and attitudes toward obesity among healthcare workers, which would facilitate comparisons across studies and enhance the reliability of future research in this area.

## Conclusion

This study demonstrates that healthcare workers continue to hold stigmatizing attitudes and misconceptions about obesity, with negative beliefs particularly common among women and older adults. These findings are consistent with international evidence, highlighting that weight stigma is a global challenge. To address this issue, healthcare systems should implement standardized educational programs, promote evidence-based understanding of the multifactorial causes of obesity, and provide compassionate, patient-centered care. Reducing weight stigma is essential for improving patient confidence, treatment adherence, and overall health outcomes. The results of this study can inform the development of more effective strategies to support individuals with obesity and foster a more inclusive and supportive healthcare environment.

## Data Availability

Due to participant confidentiality and privacy agreements, the datasets created and/or analyzed during the current study are not publicly available; however, they are available upon reasonable request from the corresponding author.

## Abbreviations

ATOP: Attitudes toward obese people
BAOP: Beliefs about obese people
BMI: Body Mass Index
BSc: Bachelor of Science
